# Natural Phenolic Inhibitors of Trichothecene Biosynthesis by the Wheat Fungal Pathogen *Fusarium culmorum*: A Computational Insight into the Structure-Activity Relationship

**DOI:** 10.1371/journal.pone.0157316

**Published:** 2016-06-13

**Authors:** Giovanna Pani, Alessandro Dessì, Roberto Dallocchio, Barbara Scherm, Emanuela Azara, Giovanna Delogu, Quirico Migheli

**Affiliations:** 1 Dipartimento di Agraria and Unità di Ricerca Istituto Nazionale di Biostrutture e Biosistemi, Università degli Studi di Sassari, Viale Italia 39, I-07100, Sassari, Italy; 2 Istituto CNR di Chimica Biomolecolare, Traversa La Crucca 3, I-07100, Sassari, Italy; Soonchunhyang University, REPUBLIC OF KOREA

## Abstract

A model of the trichodiene synthase (TRI5) of the wheat fungal pathogen and type-B trichothecene producer *Fusarium culmorum* was developed based on homology modelling with the crystallized protein of *F*. *sporotrichioides*. Eight phenolic molecules, namely ferulic acid **1**, apocynin **2**, propyl gallate **3**, eugenol **4**, Me-dehydrozingerone **5**, eugenol dimer **6**, magnolol **7**, and ellagic acid **8**, were selected for their ability to inhibit trichothecene production and/or fungal vegetative growth in *F*. *culmorum*. The chemical structures of phenols were constructed and partially optimised based on Molecular Mechanics (MM) studies and energy minimisation by Density Functional Theory (DFT). Docking analysis of the phenolic molecules was run on the 3D model of *F*. *culmorum* TRI5. Experimental biological activity, molecular descriptors and interacting-structures obtained from computational analysis were compared. Besides the catalytic domain, three privileged sites in the interaction with the inhibitory molecules were identified on the protein surface. The TRI5-ligand interactions highlighted in this study represent a powerful tool to the identification of new *Fusarium*-targeted molecules with potential as trichothecene inhibitors.

## Introduction

Trichothecene mycotoxins are produced by filamentous fungi belonging to the *Fusarium* genus and are among the major causes of crop loss in cereals [1,2]. Trichothecenes are heat resistant and very stable, and may cause severe toxicosis in mammals when contaminated grain or their derivatives are ingested. Besides affecting intestinal, immune endocrine and neurologic functions [3], trichothecenes display phytotoxic effects on the host plant, where their role in virulence has been demonstrated [4–6].

Control strategies taken so far against *Fusarium* diseases, particularly those based on the use of synthetic fungicides, are not always effective and sometimes have determined a selection pressure on fungal populations, hence facilitating the emergence of resistant mutants [7]. In this scenario, the search for alternative pest management approaches, including the development of natural fungicides or inhibitors of mycotoxin biosynthesis, appears particularly promising [8]. Natural inhibitory compounds are mostly extracted from plants and are involved in host resistance response [9].

The mechanisms by which these compounds are able to interfere with the biosynthesis of trichothecenes are not yet completely understood, and numerous hypotheses have been proposed: transcriptional control of *TRI* genes [10–11], modification of the fungal membrane permeability [12], inhibition of fungal enzymes [13], alleviation of oxidative stress that is assumed to activate the biosynthesis of mycotoxins [14]. One of the most studied interpretations implicates the inhibition of trichodiene synthase (TRI5) [11–16]. TRI5 is a dimeric sesquiterpene cyclase, encoded by the gene *TRI5*, which represents the very first step of the trichothecene biosynthesis pathway. In the presence of three magnesium (Mg^2+^) ions the TRI5 enzyme catalyses the cyclization of farnesyl pyrophosphate to form the bicyclic sesquiterpene trichodiene and the co-product inorganic pyrophosphate (PPi).

The crystal structure of TRI5 of *Fusarium sporotrichioides* with PPi bonded to the catalytic site has been isolated and highly defined by X-ray, providing a useful tool to study protein-molecule interactions with high standards [17–20]. The hypothesis assumes that the external ligand, mimicking the natural substrate of this enzyme (i.e., farnesyl pyrophosphate) binds to TRI5 inducing a conformational change of the protein such as to modify or to block its activity with the consequent decrease or total inhibition of trichothecene biosynthesis.

In a previous article [21] a collection of natural and natural-like compounds belonging to phenols and hydroxylated biphenyls was tested *in vitro* to assess their activity on vegetative growth and trichothecene biosynthesis by *F*. *culmorum*. Significant trichothecene inhibitory activity was observed over a range of concentrations comprised between 0.25–1.5 mM and some of the tested compounds proved fungitoxic.

Here we have selected eight representative compounds to test their efficacy on the *F*. *culmorum* model strain FcUK99 [22], whose full genome sequencing has been recently achieved (King, Urban, and Hammond-Kosack, *unpublished results*).

Aiming to provide further insight into the understanding of structure-activity relationship of trichothecene inhibitors and TRI5, we have adopted an approach based on modelling techniques. The crystal structure of TRI5 of *F*. *sporotrichioides* [18] was used to create a 3D atomic-level protein model of the *F*. *culmorum* TRI5 to carry out modelling and docking studies. Docking data were integrated with *in vitro* activity to identify molecular structures, functional groups and putative amino acids that are most likely involved in the interaction between selected inhibitory molecules and the *F*. *culmorum* TRI5 protein.

## Materials and Methods

### Fungal strain and culture conditions

Type B trichothecene producer wild-type strain of *F*. *culmorum* FcUK99 (Rothamsted Research, UK- NRRL54111) was used in the *in vitro* experiments. This strain produces predominantly 3-acetyldeoxynivalenol (3-ADON) and, to a lesser extent, deoxynivalenol [22]. Strain cultures were maintained as described previously by Pani *et al*., 2014 [21].

### Effect of selected phenolic compounds on *Fusarium culmorum*

**Fig 1:**
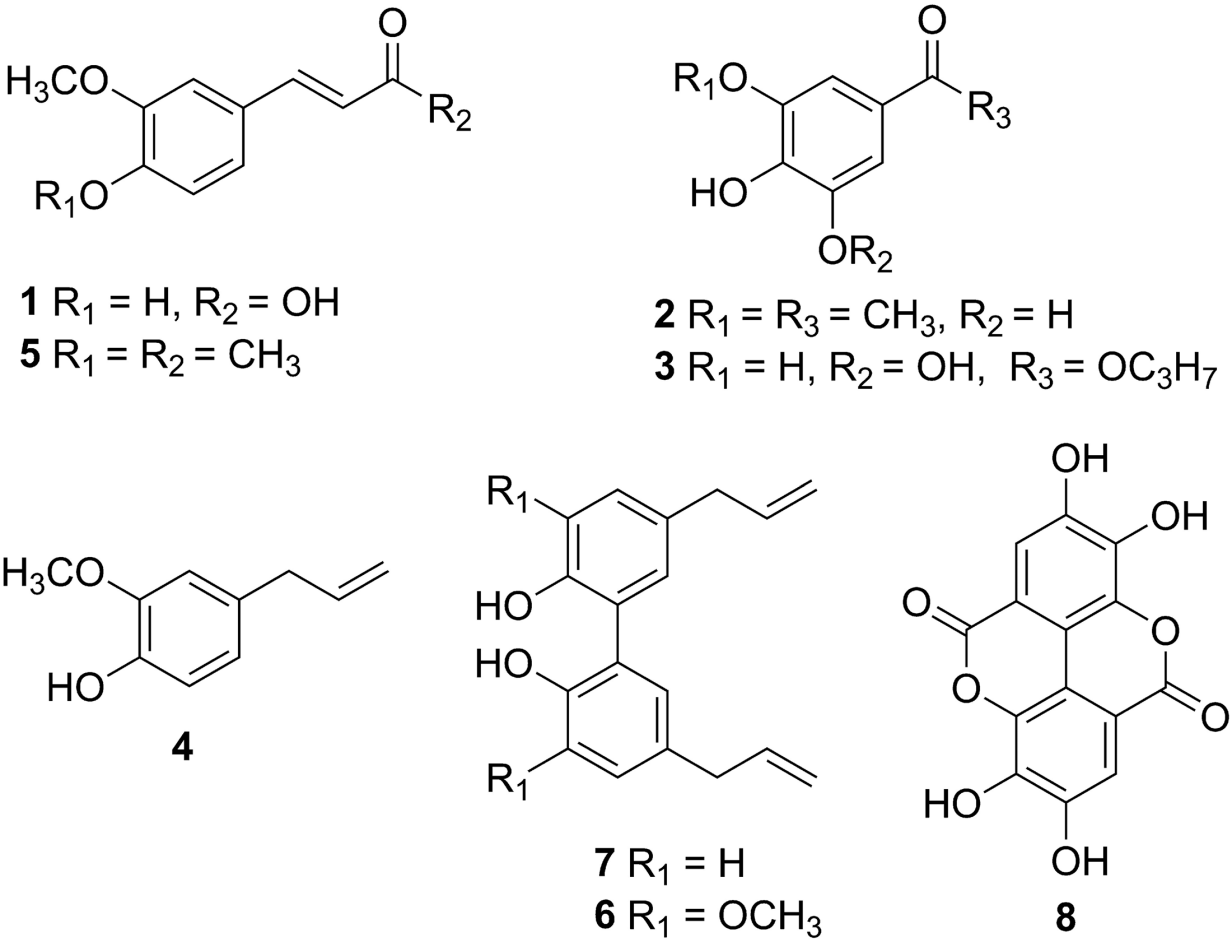
Chemical structures of tested compounds: ferulic acid 1, apocynin 2, propyl gallate 3, eugenol 4, Me-dehydrozingerone 5, eugenol dimer 6, magnolol 7, ellagic acid 8.

Ferulic acid **1**, apocynin **2**, propyl gallate **3**, eugenol **4**, Me-dehydrozingerone **5**, eugenol dimer **6**, magnolol **7**, ellagic acid **8** (Fig 1) were selected and used with a purity >98% and tested for their ability to inhibit trichothecene biosynthesis of *F*. *culmorum* strain FcUK99 at the concentration of 0.5 mM.

All compounds were purchased by Sigma-Aldrich (St. Louis, MO, USA) except for Me-dehydrozingerone **5** and eugenol dimer **6** which were prepared according to Marchiani *et al*. (2013) [23] and Dettori *et al*. (2015) [24], respectively. β-CD (CAVAMAX^®^7 PHARMA; Wacker Chemie Italia, Peschiera Borromeo, Italy) was added to the medium in order to improve solubility of phenolic molecules.

The experiments were carried out as described by Pani *et al*. (2014) [21], with some modification as follows. Each phenolic compound (0.5 mM) was added along with β-CD (3 mM) to Vogel's medium [25]. After a 60 min sonication step, the amended substrates were poured into Petri plates (60 mm diameter, 8 mL per plate, 5 replicate plates for each treatment) and inseminated with a spore suspension of strain FcUK99 (final concentration 10^4^ conidia mL^-1^). The evolution of pH in media amended with the different compounds was recorded at 0, 1, 2, 4, 8 and 14 days after inoculation (S1 Table). After 14 days incubation (at 25°C and in the dark) the culture broth and the mycelium were separated by vacuum filtration. After washing with sterile water, mycelia were harvested, dried at 80°C for 24 h and weighted. Culture broth was vortexed and an aliquot of 5 mL was collected from each replicate plate for mycotoxin extraction with ethyl acetate (3 mL), 1 min vortex, and 10 min centrifugation. Qualitative and quantitative mycotoxin detection was carried out by liquid chromatography interfaced to mass spectrometry (HP 1100, Agilent Technologies, Palo Alto, USA).

### Statistical analysis

Data on total trichothecene production (ng mL^-1^) obtained from two separate experiments were expressed as percent value of the untreated control. Data were analysed by one-way analysis of variance (ANOVA) with Dunnett’s test, using Minitab Express for Mac version 1.3.0.

### Homology modelling of TRI5

The TRI5 protein of *F*. *sporotrichioides* was prepared from the 2.50 Å resolution crystal structure deposited by Michael J. Rynkiewicz [18] (PDB code 1JFG, http://www.pdb.org). The starting TRI5 protein sequence for *F*. *culmorum* was obtained from UniProtKB/Swiss-Prot databases (accession number Q8NIG9) [26,27].

Substitution of 33 amino acids from the B chain of 1JFG was carried out with Swiss-Model, an automated comparative protein model server interconnected with Swiss-PdbViewer 4.0.4 program [28–30]. The crystallographic molecules of water and glycerol ions present in the starting TRI5 protein of *F*. *sporotrichioides* were stripped. Hydrogen atoms were added using the ADT module of MGLTools 1.5.7rc1 [31].

### Molecular docking: *in silico* studies on the binding of selected phenols to TRI5

Computational modelling experiments were conducted on multiprocessor machines with OS Ubuntu 13.04 and 14.04 or Windows 7.0. Model compounds were constructed with standard bond lengths and angles from the fragment database with MacroModel 5.5 [32]. Minimisation of structures by conformational search was performed with the MacroModel/BachMin 6.0 program using the AMBER force field. An extensive conformational search was further carried out using the Monte Carlo/energy minimisation [33] (Ei-E min <5 Kcal/mole, energy difference between the generated conformation and the current minimum). The atomic charges were assigned using the Gasteiger-Marsili method [34]. Representative minimum energy conformations of each compound were optimised using the *ab initio quantum* chemistry program Gaussian 09W by Density Functional Theory (DFT) with method B3LYP/6-311G basis set [35]. Visual analysis was carried out with GaussView version 5.0 [36]. Binding of the compounds was analysed using MGLTools 1.5.7rc1 [31] and AutoDock 4.2 docking programs [37,38]. The Gasteiger charges [34] for the ligands and proteins were used. The structures were docked using the Lamarckian genetic algorithm (LGA) defined through a centred grid (coordinates: X) -21.0, Y) 111.0, Z) 8.0, with 120, 120, 120 grid points in X, Y, Z dimensions, respectively. All ligands were docked with all bonds free to rotate. The Lamarckian genetic algorithm (LGA) of up to 100 runs was set to the population size of 150 individuals, maximum number of generations and energy evaluations of 27,000 and 15,000,000, respectively. From the estimated free energy of ligand binding (E.F.E.B., ΔG), the estimated inhibition constant (E.I.C., Ki) for each ligand was calculated. Ki is calculated by the equation: Ki = exp [(ΔG* 1000)/ (R*T)] where ΔG is the docking energy, R (gas constant) is 1.98719 cal K^−1^ mol^−1^ and T (Temperature) is 298.15 K. Graphical representation of the hypothetical positions derived from the docking calculation was obtained using the software Chimera [39].

### Estimation of selected physicochemical descriptors

Lipophilicity (LogP) of ligands was estimated with ChemBioOffice Ultra 13.0 (Perkin Elmer Inc., Waltham, M, USA), while dipole moment (D, Debye) was calculated with Gaussian 09W.

## Results

### Fungicide and inhibitory activity of phenols against *Fusarium culmorum* FcUK99

Six out of the 8 compounds (ferulic acid **1**, apocynin **2**, propyl gallate **3**, eugenol **4**, Me-dehydrozingerone **5**, and eugenol dimer **6**) demonstrated their trichothecene inhibitory activity when tested at 0.5 mM, providing a reduction in the total mycotoxin production ranging from 31% to 98%. One compound (magnolol **7**) had a fungicidal action, hence complete inhibition of mycelium growth and no trichothecene production were observed. On the contrary, ellagic acid **8** induced a significantly higher (159%) trichothecene biosynthesis compared to the untreated control (Table 1).

**Table 1:** *In vitro* effect of tested compounds on total trichothecene (DON) production by *Fusarium culmorum* FcUK99.

Compound	Dry fungal biomass	DON
	(relative yield ± SD) ^a^	(relative yield ± SD) ^b^
**1**—Ferulic acid	97.79 ± 1.40	33.62 ±19.09
**2**—Apocynin	109.48 ± 2.22	68.85 ± 4.26
**3**—Propyl gallate	94.99 ± 2.86	2.26 ± 0.75
**4**—Eugenol	93.59 ± 1.19	22.41 ± 3.35
**5**—Me-dehydrozingerone	116.57 ± 1.62	8.79 ± 2.91
**6**—Eugenol dimer	113.66 ± 1.14	11.07 ± 3.62
**7**—Magnolol	0	N.D.^c^
**8**—Ellagic acid	82.51 ± 3.68	159.50 ± 18.78

^a^ Dry fungal biomass values are expressed as mean percent values (± standard deviation) relative to the amount detected in the control culture over two separate experiments (23 ± 0.7 and 19 ± 0.7 mg, respectively). Only the treatment with magnolol is significantly different (*p*<0.001) from the untreated control based on ANOVA followed by Dunnett test.

^b^ DON yields are expressed as mean percent values (± standard deviation) relative to the amount detected in the control culture over two separate experiments (463.76 ± 120.52 and 249.17 ± 31.66 ng/mL, respectively). All values are significantly different (*p*<0.001) from the untreated control based on ANOVA followed by Dunnett test.

^c^ N.D.: not detected.

Monitoring of medium pH amended with compounds **1**–**8** showed a progressive decreasing that, after 4 days, reached a plateau around 2.2 in the presence of all compounds except for magnolol **7** that stabilised the pH at 3.38 between the 4^th^ and the 8^th^ day (S1 Table). After 14 days the medium pH was comprised between 2.41 and 2.94 for all the tested compounds.

### Computational studies: homology modelling of TRI5

As the X-ray crystal structure of *F*. *culmorum* TRI5 was not available, a 3D structure at atomic level by homology modelling was generated on the TRI5 crystal structure of *F*. *sporotrichioides* [18] and the starting protein sequence for *F*. *culmorum* (amino acid sequence code Q8NIG9). In the RCSB protein data bank (http://www.pdb.org) the trichodiene synthase of *F*. *sporotrichioides* is represented in a wide array of crystals, including wild type and mutated structures. The 3D structure homology model of *F*. *culmorum* was prepared starting from the chain B of the *F*. *sporotrichioides* crystal (1JFG), which contains the three magnesium atoms and the pyrophosphate (PPi), both essential for the enzyme activity. The model was refined by substitution of 33 amino acids from the B chain of 1JFG and by comparing the obtained result and the existent homology model sequence of *F*. *culmorum* TRI5 (Table 2).

**Table 2:** List of the amino acids modified to achieve the Q8NIG9 (*Fusarium culmorum*) compared to the 1JFGcatB crystal (*F*. *sporotrichioides*) based on the best score by Swiss-Model software.

1JFG chain B	Q8NIG9	Choosen/available^a^	Score^b^	Probability^c^	Side chain type^d^
Thr13	Ser13	R 2/3	s:-4	p:0.33	h/h
Leu55	Met5	R 6/12	s:-2	p:0.03	h/h
Lys103	Ser103	R 1/3	s:-1	p:0.55	c/c
Tyr107	His107	R 1/6	s:-3	p:0.26	h/h
Thr109	Ala109	R 1/1	s:-1	p:1.00	c/c
Val111	Leu111	R 1/4	s: 0	p:0.66	c/c
Ala124	Ser124	R 1/3	s:-2	p:0.55	c/c
Leu155	Met155	R 4/12	s:-3	p:0.21	h/h
His175	Asp175	R 7/8	s:-1	p:0.13	c/c
Glu199	Asp199	R 5/8	s:-1	p:0.07	c/c
Gln200	Leu200	R 1/4	s:-2	p:0.66	c/c
Asn202	Asp202	R 4/8	s:-5	p:0.20	c/c
Ser205	Lys205	R 1/19	s:-1	p:0.21	c/c
Leu206	Asn206	R 1/9	s:-3	p:0.33	c/c
Ser212	Thr212	R 1/2	s:-4	p:0.74	h/h
ILE214	Val214	R 1/3	s:-3	p:0.90	h/h
Tyr247	Phe247	R 3/5	s:-3	p:0.12	h/h
Val249	Thr249	R 2/2	s:-3	p:0.25	h/h
Ser250	Cys250	R 3/3	s:-4	p:0.05	h/h
Asp251	His251	R 2/6	s:-3	p:0.14	h/h
Ser254	Thr254	R 1/3	s:-4	p:0.65	c/c
His256	Asp256	R 1/4	s:-2	p:0.75	h/h
Asp265	Glu265	R 1/14	s:-3	p:0.36	h/h
Ser278	Ala278	R 1/1	s:-1	p:1.00	c/c
Arg303	Ala303	R 1/1	s:-1	p:1.00	c/c
Ser308	His308	R 4/6	s:-3	p:0.24	h/h
Glu316	Asp316	R 7/8	s:-2	p:0.13	c/c
Glu317	Gln317	R 2/15	s:-3	p:0.00	c/c
Lys318	Asp318	R 3/8	s:-2	p:0.00	c/c
Gln323	Lys323	R 1/16	s:-3	p:0.23	h/h
Tyr329	Phe329	R 1/5	s:-2	p:0.57	h/h
Ser339	Ala339	R 1/1	s:-1	p:1.00	c/c
Pro347	Gln347	R 1/15	s:-2	p:0.36	c/c

^a^ Rotamer number currently selected, followed by the number of available rotamers.

^b^ Crude score for the given rotamer. The score is obtained from: (4 x NbClash with backbone N CA and C atoms)+(3 x NbClash with backbone O atoms)+(2 x NbClash with sidechains atoms)-NbHbonds—4 x Nb SSbonds. Lower scores are preferable, while higher scores indicate clashes with the surrounding environment.

^c^ Probability to find this conformation in the current secondary structure (range from 0 to 1).

^d^ h: helix; s: strand; c:coil

Among all the possible rotamers, we focused on the mutation achieving the best score. In the crystal structure of 1JFG, residues from Arg355 to Glu374 are not resolved as in other differently resolved crystals of *F*. *sporotrichioides*. For this reason, also in the reconstruction of *F*. *culmorum* TRI5 we decided not to consider these amino acids in order to set two comparable systems.

### Docking of phenols and hydroxylated biphenyls to TRI5

**Fig 2:**
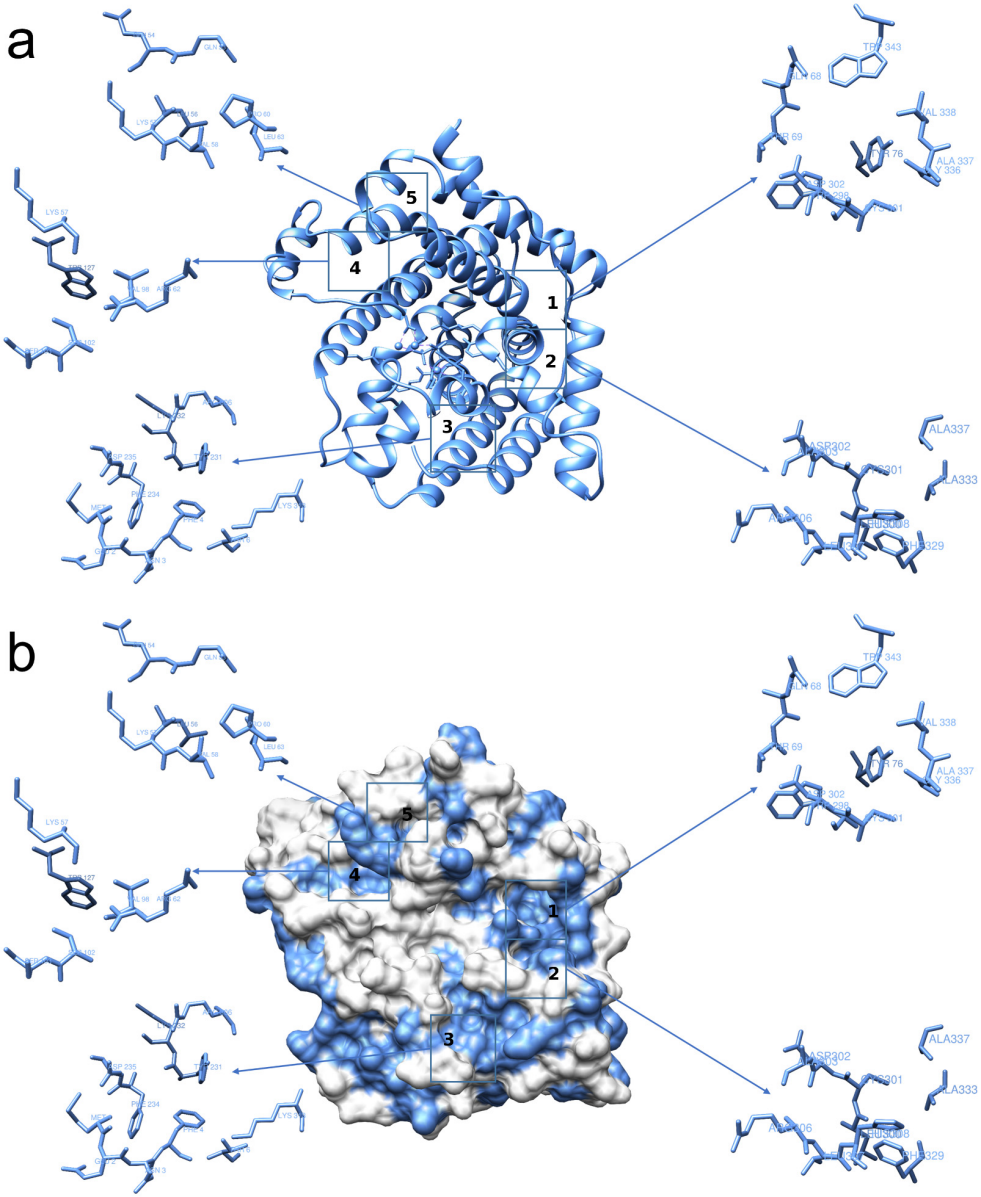
Two different representations of the interaction sites in the TRI5 protein of *Fusarium culmorum* (Q8NIG9).

All docking tests were performed considering a very large grid that included nearly the complete TRI5 enzyme of *F*. *sporotrichioides* and *F*. *culmorum*. The centre of the grid was located close to the catalytic site containing the three Mg^2+^ ions. As a result, a large number of interactions in ligands with different docking sites were evaluated. The default grid spacing (0.375 Å) was adopted, treating the docking active site as a rigid system and the ligands as flexible, i.e., all non-ring torsions were considered active and free to rotate. In addition to the active site, five further sites showing significant ligand affinities were identified on the TRI5 protein surface. These five sites, overlapping in the *F*. *sporotrichioides* and *F*. *culmorum* enzymes, are numbered from 1 to 5 (Fig 2).

A greater scattering was observed in the different sites of the *F*. *culmorum* TRI5, although the orders of magnitude of Ki and ΔG are very similar to the *F*. *sporotrichioides* TRI5 protein.

Among sequence differences (33 amino acids) occurring between the two enzymes, only four amino acids are located in the privileged binding site 2 (Lys103Ser, Arg303Ala, Ser308His, Tyr329Phe), whereas the others are in its vicinity or far away (Table 2). The difference between the four amino acids involved in the binding site has an effect on lipophilicity, slightly increasing in *F*. *culmorum* TRI5. These modifications may lead to a variation in the space available to the ligand, therefore influencing hydrophobicity, charge and polarity of the environment.

The scoring results of docking calculations deduced for ligands, as well as the major ligand receptor interactions, are listed in Table 3.

**Table 3:** Docking for protein Q8NIG9.

Tested ligands	%	Sites	E.F.E.B.^a^	E.I.C., Ki ^b^	Interactions with amino acids
Ferulic acid **1**	2	c. d.^c^	-9.62	89.12 nM	Asp100 Glu164 Leu181 Arg182 Asn185 Asp226 Glu233 Arg238 Asp239 Ser242 Leu243 Asn246 PPi700 Mg703
	6	c. d.	-7.78	1.99 uM	Ile70 Met73 Tyr93 Thr96 Asp100 Phe157 Arg182 Asn185 Leu187 PPi700 Mg703
	13	3	-5.58	80.65 uM	Met1 Glu2 Asn3 Phe4 Tyr231 Lys232 Phe234 Asp235 Arg306
	8	4	-5.51	92.16 uM	Met55 Leu56 Lys57 Arg62 Val98 Ser102 Ser103 Pro126 Trp127
	14	3	-5.48	96.45 uM	Phe4 Thr6 Tyr231 Phe234 Asp235 Arg306 Lys313
	19	5	-5.19	157.79 uM	Gln53 Gln54 Leu56 Lys57 Val58 Pro60 Leu63
Apocynin **2**	4	c. d.	-6.86	9.29 uM	Asp100 Glu164 Arg182 Asn185 Asp226 Glu233 Arg238 Asp239 Ser242 Leu243 PPi700 Mg703
	60	1	-5.35	119.64 uM	Gln68 Tyr76 Cys301 Asp302 Gly336 Ala337 Val338 Trp343
	11	1	-5.25	134.28 uM	Gln68 Tyr69 Gly72 Tyr76 Trp298 Cys301 Asp302 Gly336 Ala337 Val338
Propyl gallate **3**	40	1	-5.86	50.71 uM	Ala65 Gln68 Thr69 Tyr76 Trp298 Cys301 Asp302 Ala303 Gly336 Val338 Pro340 Trp343
	13	2	-5.28	133.84 uM	Leu300 Cys301 Asp302 Ala303 Arg306 Leu307 His308 Phe329 Ala333 Ala337
Eugenol **4**	3	c. d.	-5.65	72.36 uM	Asp100 Glu164 Asn181 Arg182 Asn185 Asp226 Glu233 Arg238 Asp239 Ile241 Ser242 Leu243 Asn246 PPi700
	31	2	-5.12	177.48 uM	His299 Leu300 Cys301 Asp302 Ala303 Arg306 Leu307 His308 Glu309 Phe329
	19	2	-5.07	193.10 uM	His299 Leu300 Cys301 Asp302 Ala303 Arg306 Leu307 His308 Phe329 Ala333 Ala337
	22	1	-4.98	224.72 uM	Gln68 Thr69 Tyr76 Trp298 Cys301 Asp302 Gly336 Ala337 Val338 Trp343
Me-Dehydrozingerone **5**	14	4	-5.85	51.63 uM	Met55 Leu56 Lys57 Arg62 Val98 Ser102 Ser103 Pro126 Trp127
	22	2	-5.27	139.91 uM	His299 Leu300 Cys301 Asp302 Ala303 Arg306 Leu307 His308 Glu309 Phe329
	32	2	-5.20	153.52 uM	Leu300 Cys301 Asp302 Ala303 Arg306 Leu307 His308 Phe329 Ala333
Eugenol dimer **6**	14	1–2	-6.69	12.41 uM	Gln68 Gly72 Tyr76 Trp298 Cys301 Asp302 Ala303 His308 Ala337 Val338
	28	1–2	-6.67	12.98 uM	Thr69 Gly72 Tyr76 Trp298 Leu300 Cys301 Asp302 Ala303 Phe329 Ala333 Ala337 Val338 Ala339 Pro340 Trp343
	10	1–2	-6.27	25.41 uM	Leu36 Gln68 Thr69 Tyr76 Leu300 Cys301 Asp3Ala303 Phe329 Ala333 Gly336 Ala337 Val338 Trp343
	14	4	-5.41	108.76 uM	Lys57 Val58 Arg62 Val98 Leu99 Ser102 Ser103 Asp104 Pro126 Trp127
Magnolol **7**	29	1	-6.91	8.56 uM	Gln68 Thr69 Gly72 Tyr76 Leu300 Cys301 Asp302 Phe329 Gly336 Ala337 Val338 Pro340
	11	1–2	-6.49	17.43 uM	Leu36 Gly72 Tyr76 Trp298 Leu300 Cys301 Asp302 Ala303 Phe329 Ala333 Ala337 Val338 Pro340 Trp343
	14	4	-5.91	46.77 uM	Met55 Leu56 Lys57 Val58 Arg62 Val98 Ser102 Ser103 Pro126 Trp127
Ellagic acid **8**	20	4	-7.00	7.44 uM	Met55 Leu56 Lys57 Arg62 Val98 Ser102 Ser103 Pro126
	36	1	-6.68	12.64 uM	Ala65 Gln68 Thr69 Gly72 Tyr76 Asp302 Ala303 Gly336 Val338 Pro340 Trp343
	27	2	-6.18	29.44 uM	Leu300 Cys301 Asp302 Ala303 Arg306 Leu307 His308 Phe329 Ala333

^a^ E.F.E.B.: Estimated Free Energy of Binding.

^b^ E.I.C., Ki: Estimated Inhibition Constant, Ki.

^c^ c. d.: catalytic domain.

**Fig 3:**
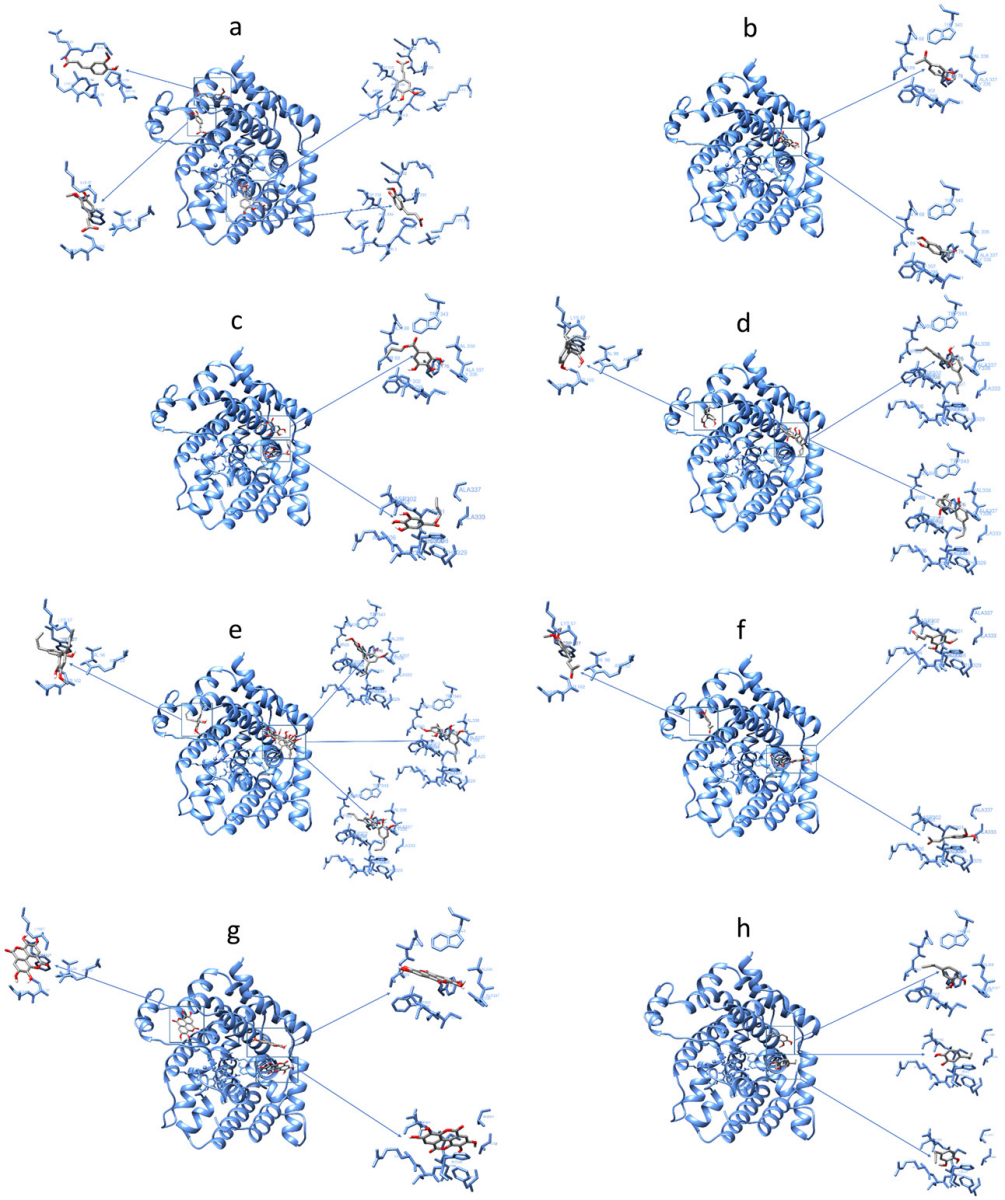
Interaction sites between the TRI5 protein of *Fusarium culmorum* model and the tested compounds. (**a**) ferulic acid; (**b**) apocynin; (**c**) propyl gallate; (**d**) magnolol; (**e**) eugenol dimer; (**f**) Me-dehydrozingerone; (**g**) ellagic acid; (**h**) eugenol.

The ligands can be clustered into three main groups: (i) molecules characterised by a carboxyl group, likely deprotonated at physiological pH, and having a charge of -1 (compound **1**); (ii) molecules with charge 0 (compounds **2**, **3**, **4**, **5**); and (iii) dimeric molecules (compounds **6**, **7**, **8**). Docking studies indicate that sites 1, 2, and 4 of *F*. *culmorum* Q8NIG9 are privileged sites for ligands inhibiting trichothecene biosynthesis *in vitro*. Ligands with a long aliphatic chain and dimeric molecules (hydroxylated biphenyls) interact simultaneously with sites 1 and 2 (Fig 3).

**Fig 4:**
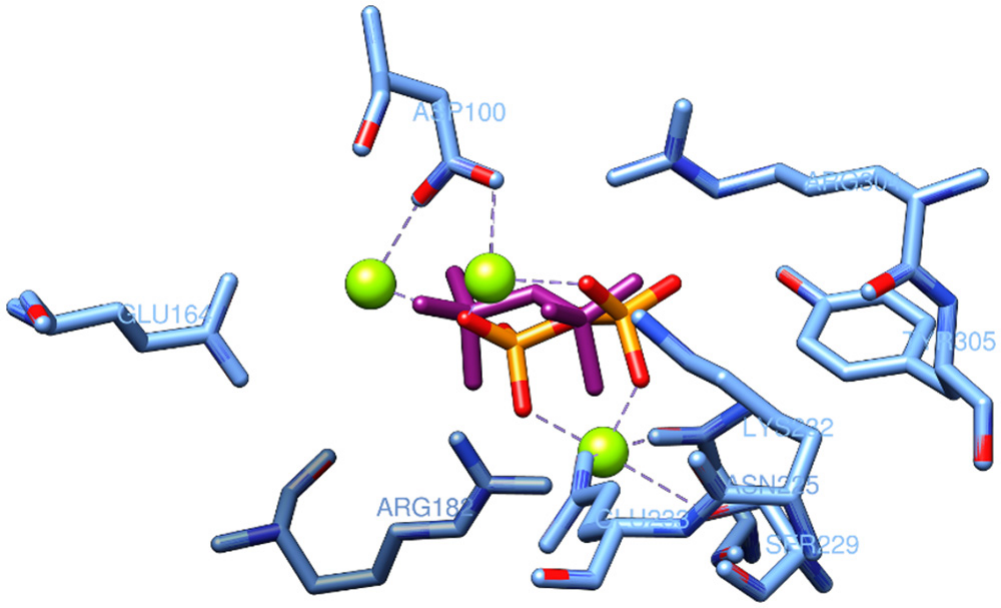
Pyrophosphate positions in the catalytic site of the X-ray structure (violet) and in the model (orange) of the TRI5 protein.

In docking calculations, PPi was always considered as an additional fixed residue. The reliability of the docking approach was further verified by extracting the PPi from the catalytic site and by considering it as a normal ligand. After repositioning of PPi into the protein, the new PPi location was the same as in the original X-ray structure of TRI5, with only minimal conformational changes (RMSD 1.27 Å), hence confirming the reliability of the system (Fig 4).

Ferulic acid **1**, the sole charged phenol among the eight ligands tested in *in vitro*, interacts with the catalytic site in the proximity of the Mg^2+^ ions with significant docking score (Ki 1.99 μM) and with amino acids of three sites located in the surface of TRI5, identified as site 3, 4 and 5 (Fig 3a; Table 3). The estimated free energy of binding (E.F.E.B.) at sites 3, 4 and 5 was calculated over a range between -5.19 and -5.58 Kcal/mol (Table 3).

**Fig 5:**
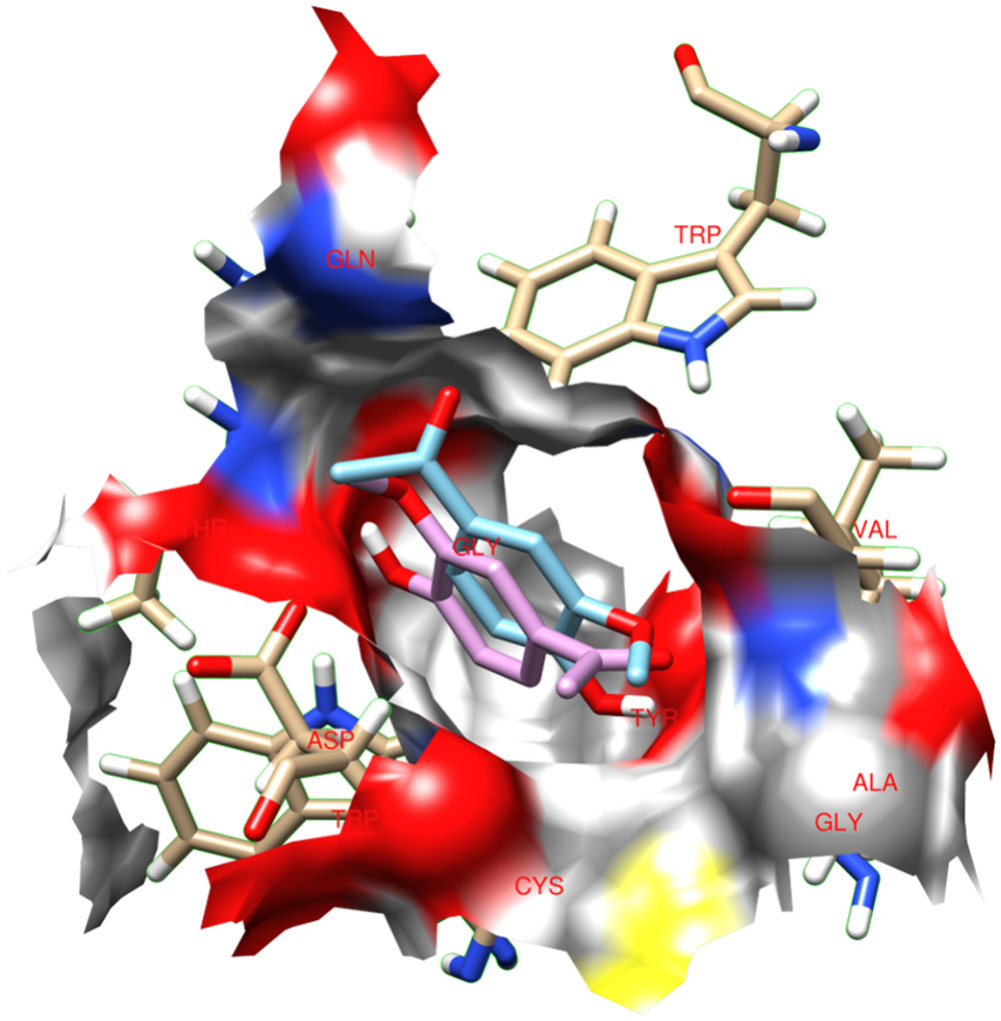
Interaction between apocynin 2 and the site 1 of the TRI5 protein. Coloured areas represent the atoms of the different interacting amino acids.

Generally, monomers interact with sites 1 and 2, favouring site 1. Apocynin **2**, which inhibited trichothecene biosynthesis by approximately 30% in *in vitro* assays, displayed 71% of docking runs in site 1 (Table 3), with an energy of -5.35 Kcal/mole and Ki of 119.64 μM. In Fig 5, the coloured areas represent the atoms of the different amino acids interacting with apocynin **2**. In virtue of its structural and chemical functional features, the apocynin molecule is fully positioned within the cavity formed by these amino acids.

Docking experiments with propyl gallate **3** (trichothecene production reduced by 98%) showed a similar behaviour. Forty per cent of docking runs occurred at the lowest calculated energy of -5.86 Kcal/mole and Ki 50.71 μM (Table 3). Furthermore, propyl gallate **3** fits in 13% of the docking runs (-5.28 Kcal/mol) to site 2 with a Ki 133.84 μM (Table 3).

Eugenol **4** (able to reduce trichothecene production by approximately 78%), binds in 22% of the cases to site 1 of the TRI5 protein (-4.98 Kcal/mole). Despite the low inhibition constant Ki (224.72 μM) calculated for site 1, eugenol **4** binds also (50% of docking run sum) to site 2, with two different conformations. Eugenol **4** shows affinity for this site with energies of -5.12 Kcal/mole and -5.07 Kcal/mole, according to its conformations. Eugenol **4** and apocynin **2** interact also with the catalytic domain with negligible docking run, therefore, these interactions were ruled out.

Me-dehydrozingerone **5**, a molecule that inhibited 92% of the trichothecene production, interacts with site 2 with two distinct conformations of similar energy with a docking run sum of 54%. Moreover, Me-dehydrozingerone **5** also interacts with site 4 with 14% of docking run and significant Ki (51.63 μM) and estimated free energy of binding (-5.85 Kcal/mol).

Eugenol dimer **6**, magnolol **7** and ellagic acid **8** are dimeric phenols belonging to the family of hydroxylated biphenyls. These dimeric molecules are structurally different from the other two groups as they display a more hindered structure due to the presence of two aromatic rings linked by a single C-C bond. Two lactone groups fused in the C-C single bond make the structure of ellagic acid **8** quite planar, whereas eugenol dimer **6** and magnolol **7** may freely rotate along with the C-C single bond. These latter two ligands enter in site 1 with one of their two aromatic rings, positioning the second one perpendicularly to the first one that then interacts with the amino acids located in site 2, in proximity to site 1. This conformation allows magnolol **7** and eugenol dimer **6** to interact simultaneously with the amino acids of site 1 and 2 by each aromatic ring. Magnolol **7** interacts with sites 1 and 2 with two conformations that are quite equal in energy (29% and 11% of docking runs, respectively), and with site 4 in 14% of the docking runs. The energy ranges between -6.91 and -5.91 Kcal/mole with significant Ki up to 8.56 μM (Table 3). The eugenol dimer **6** behaves similarly to magnolol **7**, binding at the same time to sites 1 and 2 with a probability of 52% as sum of the three conformations similar in energy, while interactions with site 4 were assessed to 14% (Table 3).

On the contrary, ellagic acid **8** (the sole dimeric compound inducing a significant increase in the production of trichothecene by *F*. *culmorum* FcUK99 when added to the substrate at the tested concentration; Table 1), interacts separately and with significant Ki with site 1 (36% of docking runs) and site 2 (27% of docking runs; Table 3). The lowest energy (-7.00 Kcal/mole) and the best Ki (7.44 μM), respectively, were predicted for ellagic acid **8** when interacting with site 4 (Table 3). The flat conformation of ellagic acid **8** forces the molecule to assume an intercalating position in the groove.

## Discussion

A wide array of natural and natural-like phenols and dimers belonging to cinnamic acids, acetophenones, benzaldehydes, benzoic acids, phenylpropanoids, and hydroxylated biphenyls has already proven able to inhibit vegetative growth and/or trichothecene production when tested on a 3-acetyl-4-deoxynivalenol producing strain of *F*. *culmorum* [21]. In our previous study, no linear correlation was observed between antioxidant properties of the tested compounds and their inhibitory effect on fungal growth and mycotoxin production. Nonetheless, a guaiacyl unit in the structure was provisionally hypothesized to play a key role in trichothecene inhibition.

In the present investigation, we aimed to provide further insight into the understanding of structure-activity relationship of trichothecene inhibitors and the trichodiene synthase protein TRI5. Hence, we selected eight compounds based on their structural properties and on their inhibitory activity towards the *F*. *culmorum* wild-type strain FcUK99, already used in a number of genomics and biological studies [22,40,41].

The tested compounds ferulic acid **1**, apocynin **2**, propyl gallate **3**, eugenol **4** and Me-dehydrozingerone **5** are phenols, whereas ellagic acid **8**, magnolol **7** and eugenol dimer **6** are C_2_-symmetry hydroxylated biphenyls featured by two aromatic rings bonded by a single C-C bond (Fig 1).

Preliminary docking experiments with selected phenols onto the *F*. *culmorum* TRI5 protein model predicted that these compounds occupy five binding pockets that are different from the catalytic site. Sites 1, 2, and 4 are privileged sites of no-charged ligands where non-covalent interactions would take place, whereas ferulic acid **1** activates interactions with the catalytic domain with significant Ki, preferentially with magnesium atoms and the PPi, although non-covalent interactions with amino acids of sites 3, 4 and 5 were also estimated with significant docking score (Table 3).

In the tested experimental conditions, all compounds, among which ferulic acid **1** presents the lowest pKa (4.48), are likely to be in the protonated form, as confirmed by the low pH value detected in the medium since the first days of fungal growth.

Propyl gallate **3**, ellagic acid **8**, magnolol **7**, eugenol **4**, and the eugenol dimer **6** bound preferentially to sites 1 and 2 located on the surface of TRI5 and far from the catalytic domain (Fig 3). With few exceptions or to a different extent, no-charged phenols interact with the same set of amino acids identified as: Gln68, Thr69, Tyr76, Trp298, Leu300, Cys301, Asp302, Ala303, His308, Phe329, Ala333, Gly336, Ala337, Val338, Trp343. Except for Asp302, these amino acids, that are most involved in the protein-ligand interactions, are hydrophobic, hence lipophilicity of phenols may play an important role in trichothecene synthase inhibition [42,43].

Magnolol **7** and eugenol dimer **6** bind to sites 1 and 2 by partially or totally overlapping, in virtue of their conformational flexible structure that provides the best interaction with the set of surrounding amino acids. The best docking pose for biphenyls **7** and **8** included one aromatic moiety of biphenyl engaging amino acids that are present in site 1, while the other aromatic moiety interacts with amino acids in site 2. In the case of ellagic acid **8**, though, significant docking runs were calculated (accounting for 36% and 27% for site 1 and 2, respectively): due to its almost flat structure, ellagic acid **8** does not overlap with sites 1 and 2, exerting a less intimate contact when compared with conformationally flexible magnolol **7** and eugenol dimer **6**. These two molecules also bind to site 5 with a significant docking (E.F.E.B.) (Table 3; Fig 3d and 3e). It is known that hydroxylated biphenyls are privileged molecules for protein binding in comparison to other aromatic compounds [44]. Such behaviour derives from the flexible structure of the biphenyl unit that can be accommodated, with high level of specificity, within a wide variety of protein pockets. The presence of a C_2_ symmetry axis in the hydroxylated biphenyls **6**–**8** makes the two aromatic rings indistinguishable from the chemical and biological point of view [24,45,46]. This structural feature facilitates the docking, reducing the number of possible interactions between the two parts of the molecule because they are identical.

Propyl gallate **3**, which presents an aliphatic side chain, activates interactions with a large number of hydrophobic amino acids that are common to magnolol **7** and eugenol dimer **6** (Thr69, Trp298, Leu300, Cys301, Ala303, Phe329, Ala333, Ala337, Trp343), confirming the high inhibitory activity observed *in vitro*. To a lesser extent compared to the other sites, Me-dehydrozingerone **5**, magnolol **7**, eugenol dimer **6** and ellagic acid **8** activate non-covalent interactions with a common set of amino acids located in site 4 (Lys57, Arg62, Val98, Ser102, Ser103, Pro126 and Thp127).

The energetically “best” poses for apocynin **2** involve only site 1 with significant docking run values, whereas Me-dehydrozingerone **5** engages sites 4 and 2, the latter with almost equal docking runs in two molecule conformations. We hypothesize that the far less efficient trichothecene inhibition observed in the case of apocynin **2** may be related to its limited number of non-covalent interactions with TRI5, whereas a large number of amino acids are involved in the interaction between TRI5 and Me-dehydrozingerone **5**, which activates interactions with the hydrophobic amino acids present in site 2.

Another factor evidenced in the docking studies is the capacity of ligands with high trichothecene inhibitory properties to activate hydrogen bond (H-bond) interactions with amino acids of TRI5 (Table 4). Biphenyls **6**–**8** establish multiple H-bonds and hydrophobic interactions with the amino acids present in sites 1, 2 and 4 (Table 4; Fig 3d, 3e and 3g). Cross-bridge interactions with the same amino acids and the OH-phenol group of ferulic acid **1**, propyl gallate **3**, eugenol dimer **6**, magnolol **7** and ellagic acid **8** were predicted (Table 4).

**Table 4:** H-bond interaction of tested ligands-protein, logP and Dipole Moment of the ligands.

Tested ligands	%	Sites	Hbond	Ligands Atom	Protein Atom	Distance (Å)^a^	LogP	Dipole Moment (D)
Ferulic acid **1**	2	c. d.^b^	5	O12(OA)	Asn185:2HD2(HD)	2.118	1.70	26.467
				H15(HD)	Asp226:OD1(OA)	1.887		
				O11(OA)	Arg238:HE(HD)	**2.299**		
				O11(OA)	Arg238:1HH2(HD)	**2.196**		
				O14(OA)	Leu243:HN(HD)	1.985		
	6	c. d.	0	-----------	---------------------	--------		
	13	3	4	H15(HD)	Glu2:O(OA)	2.023		
				O14(OA)	Phe4:HN(HD)	2.093		
				O10(OA)	Asp235:HN(HD)	1.925		
				O11(OA)	Arg306:1HH2(HD)	1.779		
	8	4	5	H15(HD)	Met55:O(OA)	2.269		
				O12(OA)	Lys57:HN(HD)	2.147		
				O10(OA)	Arg62:1HH1(HD)	**1.929**		
				O10(OA)	Arg62:1HH2(HD)	**2.235**		
				O11(OA)	Ser103:HN(HD)	2.208		
	14	3	7	O11(OA)	Thr6:HG1(HD)	2.592		
				H15(HD)	Tyr231:O(OA)	1.860		
				O14(OA)	Asp235:HN(HD)	2.276		
				O12(OA)	Arg306:1HH2(HD)	**2.481**		
				O12(OA)	Arg306:2HH2(HD)	**2.391**		
				O10(OA)	Lys313:HZ3(HD)	**1.946**		
				O11(OA)	Lys313:HZ2(HD)	**1.809**		
	19	5	3	O14(OA)	Gln53:2HE2(HD)	2.271		
				O10(OA)	Lys57:HZ3(HD)	**2.483**		
				O11(OA)	Lys57:HZ2(HD)	**1.718**		
Apocynin **2**	4	c. d.	3	O10(OA)	Asn185:2HD2(HD)	2.087	0.83	5.650
				H13(HD)	Asp226:OD1(OA)	1.828		
				O12(OA)	Leu243:HN(HD)	1.846		
	60	1	4	O9(OA)	Gln68:1HE2(HD)	2.163		
				O12(OA)	Tyr76:HH(HD)	2.386		
				H13(HD)	Gly336:O(OA)	1.977		
				O10(OA)	Val338:HN(HD)	2.043		
	11	1	3	H13(HD)	Gln68:O(OA)	2.172		
				O12(OA)	Trp298:HE1(HD)	2.305		
				O9(OA)	Val338:HN(HD)	1.822		
Propyl gallate **3**	40	1	7	O8(OA)	Gln68:1HE2(HD)	**2.243**	1.51	4.534
				O9(OA)	Gln68:1HE2(HD)	**2.280**		
				H18(HD)	Tyr76:OH(OA)	2.395		
				H16(HD)	Asp302:OD2(OA)	2.086		
				H14(HD	Gly336:O(OA)	**2.134**		
				H18(HD)	Gly336:O(OA)	**2.244**		
				O13(OA)	Val338:HN(HD)	1.932		
	13	2	5	H14(HD)	Asp302:O(OA)	**1.926**		
				H18(HD)	Asp302:O(OA)	**2.005**		
				H18(HD)	Ala303:O(OA)	2.591		
				O17(OA)	Arg306:HN(HD)	2.253		
				O13(OA)	Leu307:HN(HD)	1.979		
Eugenol **4**	3	c. d.	3	O10(OA)	Asn185:2HD2(HD)	1.919	2.57	1.372
				H13(HD)	Asp226:OD1(OA)	1.819		
				O12(OA)	Leu243:HN(HD)	2.318		
	31	2	1	H13(HD)	Leu300:O(OA)	2.003		
	19	2	2	H13(HD)	Asp302:O(OA)	2.204		
				O10(OA)	Leu307:HN(HD)	1.977		
	22	1	2	H13(HD)	Gly336:O(OA)	2.031		
				O12(OA)	Val338:HN(HD)	1.845		
Me-dehydrozingerone **5**	14	4	3	O12(OA)	Lys57:HN(HD)	2.174	1.53	5.123
				O10(OA)	Arg62:1HH1(HD)	2.185		
				O10(OA)	Ser103:HN(HD)	2.565		
	22	2	0	------------	---------------------	--------		
	32	2	1	O10(OA)	Leu307:HN(HD)	1.875		
Eugenol dimer **6**	14	1–2	3	O15(OA)	Gln68:1HE2(HD)	2.099	4.78	0.172
				O20(OA)	Val338:HN(HD)	**2.176**		
				H21(HD)	Val338:O(OA)	**2.163**		
	28	1–2	2	O25(OA)	Ala303:HN(HD)	2.166		
				H14(HD)	Val338:O(OA)	2.187		
	10	1–2	3	H21(HD)	Tyr76:OH(OA)	2.458		
				H21(HD)	Gly336:O(OA)	2.141		
				O20(OA)	Val338:HN(HD)	1.991		
	14	4	4	H14(HD)	Lys57:O(OA)	2.019		
				O13(OA)	Arg62:1HH2(HD)	1.881		
				O20(OA)	Ser103:HN(HD)	**1.827**		
				H21(HD)	Ser103:OG(OA)	**2.155**		
Magnolol **7**	29	1–2	3	O18(OA)	Tyr76:HH(HD)	2.482	5.03	3.655
				H19(HD)	Gly336:O(OA)	1.907		
				H17(HD)	Val338:O(OA)	1.889		
	11	1–2	3	H17(HD)	Cys301:O(OA)	2.167		
				O16(OA)	Ala303:HN(HD)	2.412		
				H19(HD)	Val338:O(OA)	1.993		
	14	4	4	O16(OA)	Arg62:1HH2(HD)	1.906		
				H17(HD)	Ser102:OG(OA)	**2.270**		
				H19(HD)	Ser102:OG(OA)	**1.880**		
				O18(OA)	Ser103:HN(HD)	2.091		
Ellagic acid **8**	20	4	9	H20(HD)	Met55:O(OA)	**2.134**	1.05	0.001
				H22(HD)	Met55:O(OA)	**2.192**		
				O19(OA)	Lys57:HN(HD)	2.581		
				O18(OA)	Arg62:1HH2(HD)	2.020		
				O8(OA)	Ser102:HG(HD)	2.406		
				O23(OA)	Ser103:HN(HD)	**2.026**		
				H24(HD)	Ser103:OG(OA)	**2.139**		
				H26(HD)	Ser103:O(OA)	**2.193**		
				H26(HD)	Ser103:OG(OA)	**1.837**		
	36	1	6	H22(HD)	Thr69:OG1(OA)	1.900		
				O25(OA)	Tyr76:HH(HD)	2.236		
				H26(HD)	Gly336:O(OA)	1.993		
				O23(OA)	Val338:HN(HD)	**2.163**		
				H24(HD)	Val338:O(OA)	**1.850**		
				O25(OA)	Val338:HN(HD)	**2.195**		
	27	2	3	H24(HD)	Leu300:O(OA)	1.795		
				H20(HD)	Ala303:O(OA)	1.721		
				O18(OA)	Leu307:HN(HD)	2.345		

^a^ Cross-bridge H-bond interactions with the same aa are listed in bold.

^b^c. d. = catalytic domain.

Cross-bridge H-bond interactions of biphenyls **6**–**8** and propyl gallate **3** involve mostly apolar and hydrophobic amino acids (Table 4), whereas polar and less hydrophobic amino acids activate interactions with ferulic acid **1**. This type of interaction was not observed for eugenol **4**, apocynin **2** and Me-dehydrozingerone **5**, although the latter ligand interacts with sites 2 and 4 with a large number of hydrophobic amino acids.

Overall, the docking results confirmed what we had observed in a previous *in vitro* assay with a far larger collection of phenols [21]: lipophilicity and H-bonding capacity were postulated as key factors in the selection of a “good” trichothecene inhibitor. Although no π-π-stacking interactions between protein-ligands were predicted, these would not be ruled out.

Furthermore, the molecular size (i.e., the capacity of the ligand to interact with different amino acids at the same time) should be taken into account [47,48]. From this perspective, the scarce inhibitory activity of apocynin **2** might reflect its relatively small size, which limits extensive protein-ligand interactions.

As mentioned before, sites 1, 2 and 4 were identified as privileged sites of no-charged phenols. Quite interestingly, five amino acids (Cys301, Asp302, Ala303, Phe329, and Ala337) are represented in the range of 80–100% in sites 1 and 2, where most of the ligand-protein docking occurs. These amino acids have neutral and, except for Asp302, non-polar side chains, assuming a lipophilic character of the potentially binding ligand. A prevalence of hydrophobic amino acids also resulted in site 4.

To sum up, propyl gallate **3**, Me-dehydrozingerone **5**, eugenol dimer **6** and magnolol **7** proved the best trichothecene inhibitors, as predicted by docking studies, where these compounds bind to the same sites of TRI5, and further confirmed by the *in vitro* bioassay. The reduced inhibitory activity observed *in vitro* for apocynin **2**, eugenol **4** and ellagic acid **8** might be related to restricted ligand-protein interaction.

As mentioned, magnolol **7** behaved as a fungicide *in vitro* when tested at 0.5 mM concentration. However, in the modelling study, ligand concentrations were not taken into account, and magnolol **7** may therefore switch from fungicide to trichothecene inhibitor at very low concentration, as shown previously [21]. The fungicidal activity of magnolol **7** should be further investigated since its properties are likely to affect other mechanisms of action related to both primary and secondary metabolism [49].

**Fig 6:**
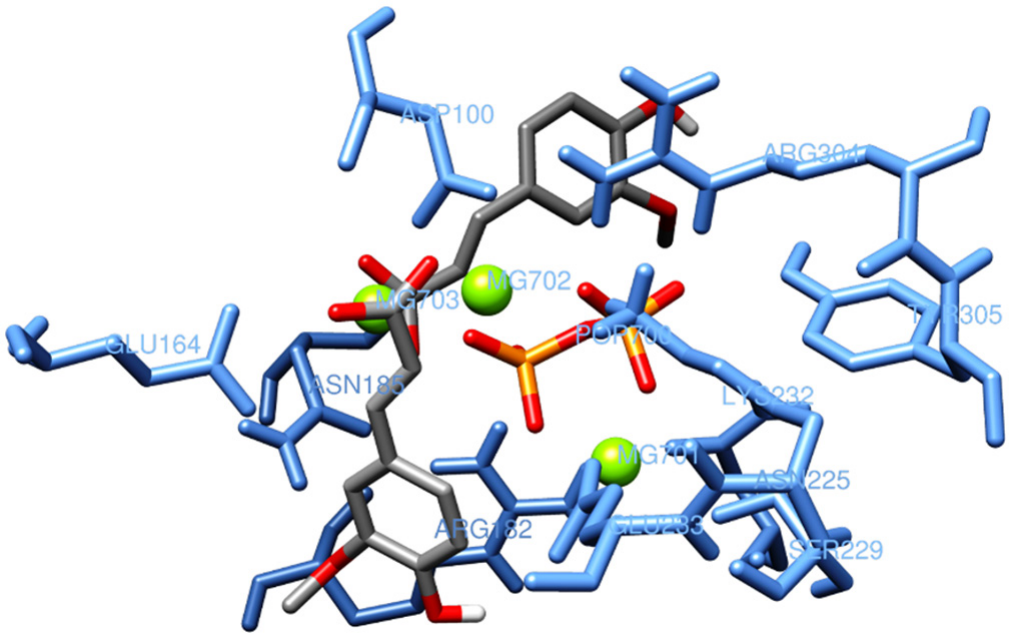
Interaction between ferulic acid 1 and the catalytic domain of the TRI5 protein.

Ferulic acid **1** is a cinnamic acid and was included in this study due to its well-known inhibitory activity towards trichothecene biosynthesis [50,51]. This compound demonstrated a high affinity with the catalytic domain of the TRI5 protein by strong metal- and ion-interactions (Fig 6), whereas the best docking scores were estimated with amino acids of sites 3, 4 and 5 where ferulic acid **1** likely activates non-covalent interactions.

Aiming to further investigate the interaction of *F*. *culmorum* TRI5 with phenolic acids, we compared the docking score of ferulic acid **1** with other cinnamic acids and benzoic acids whose biological activity has been previously tested *in vitro* [21]. 3-Hydroxycinnamic acid **9**, 4-hydroxycinnamic acid **10**, 2,5-dimethoxycinnamic acid **11** inhibited trichothecene production by *F*. *culmorum* strain MCf 21 (syn. INRA 117) in the range of 1.5 and 1.0 mM, whereas no trichothecene nor mycelium growth inhibition were observed for both 3-methoxybenzoic acid **12** and 3,4-dimethoxybenzoic acid **13** at 0.5 mM [21]. All phenolic acids interact significantly with the catalytic domain with higher docking scores compared to ferulic acid **1** (S2 Table). Contrary to benzoic acids **12** and **13**, all cinnamic acids interact with sites 3, 4 and 5 as estimated in ferulic acid **1** evidencing an important role of the α,β-unsaturated chain in activating interactions with sites located on the protein surface. Substituents in the aromatic ring of cinnamic acids are likely to influence H-bond interactions, that increase when the hydroxyl group is in *para* position to the aliphatic chain, hence favouring electron delocalization as in compounds **1** and **10** (S3 Table). Nevertheless, ferulic acid **1** accounts for the highest H-bonds involving amino acids located on protein surface. Site 3 and, to a lesser extent, site 5 seem to be privileged sites for cinnamic acids (charged-phenols), mostly binding to polar and partially hydrophobic amino acids.

## Conclusions

In the present study, a docking method was applied to explore and validate key interactions between natural and natural-like phenols with the *F*. *culmorum* TRI5 model. Apart from the catalytic domain, five binding sites were identified on the surface of TRI5 and among them sites 1, 2 and 4 are privileged binding sites for no-charged phenols. For charged phenols, and particularly ferulic acid **1** which was investigated as a prototype of cinnamic acids, sites 3 and 5 are privileged for binding, despite interaction with the catalytic domain should be also considered for more polar cinnamic acids.

Docking data confirmed results obtained from *in vitro* assays with a collection of phenols, whose lipophilicity and H-bonding capacity were postulated as key factors in the selection of a good trichothecene inhibitor. An important role might be played by the molecular size, which governs the ability of the ligand to interact with different amino acids at the same time.

Hence, our model assumes that a phenolic molecule bearing substituents with high ability to activate protein-ligand interactions at the same time with a large number of amino acids would be an ideal candidate as trichothecene inhibitor.

It has to be born in mind that the modelling approach provides only an estimation of protein-ligand interaction, as it does not take into consideration a wide range of interfering factors (e.g., concentration, interactions with other proteins, interactions with water molecules, delivery system, membrane permeability, biocompatibility and bioavailability). Nonetheless, the TRI5-ligand interactions highlighted in this study, along with the availability of a crystal structure of the *Fusarium* TRI5, shall provide an additional tool to discover new molecules with potential as fungicides or as trichothecene inhibitors, and will guide the synthesis of novel *Fusarium*-targeted compounds by shortening the time of research and by reducing cost. The long-term goal of our project is to develop natural or natural-like phenolic compounds as effective, environmentally-friendly alternatives to synthetic fungicides. Based on *in vitro* and *in silico* characterisation, field testing is now being carried out with selected molecules in order to evaluate their efficacy in reducing FHB symptoms in durum wheat as well as trichothecene contamination in harvested grain.

## Supporting Information

S1 Fig Chemical structures of tested compounds: Ferulic acid 1, 3 Hydroxycinnamic acid 9, 4- Hydroxycinnamic acid 10, 2,5-Dimethoxycinnamic acid 11, 3-Methoxybenzoic acid 12, 3,4- Dimethoxycinnamic acid 13. (TIF)

S1 Table Evolution of pH in Vogel’s medium amended with different phenolic compounds during 0–14 days after inoculation with *F*. *culmorum* strain FcUK99. (DOCX)

S2 Table Docking for protein Q8NIG9. (DOCX)

S3 Table H-bond interaction of tested ligands-protein, logP and Dipole Moment of the ligands. (DOCX)
