# Supplementary figures and images for: Natural Phenolic Inhibitors of Trichothecene Biosynthesis by the Wheat Fungal Pathogen Fusarium culmorum: A Computational Insight into the Structure-Activity Relationship
Source: PLoS One. 2016 Jun 13;11(6):e0157316. doi: 10.1371/journal.pone.0157316 (PMC4905666; doi:10.1371/journal.pone.0157316)

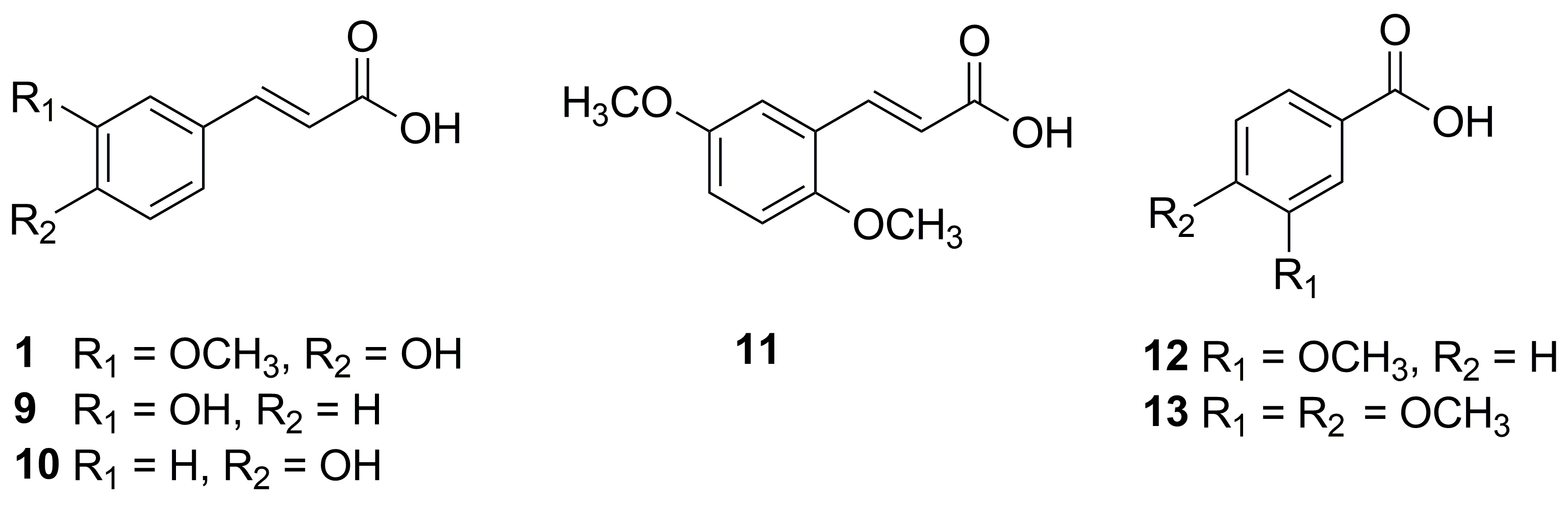

Supplement: S1 Fig — (TIF) [file pone.0157316.s001.tif]
