# Supplementary material for: Natural Phenolic Inhibitors of Trichothecene Biosynthesis by the Wheat Fungal Pathogen Fusarium culmorum: A Computational Insight into the Structure-Activity Relationship
Source: PLoS One. 2016 Jun 13;11(6):e0157316. doi: 10.1371/journal.pone.0157316 (PMC4905666; doi:10.1371/journal.pone.0157316)
Supplement: S1 Table — (DOCX) [file pone.0157316.s002.docx]

| **Compound** | **0** | **1 d** | **2 d** | **4 d** | **8 d** | **14 d** |
| --- | --- | --- | --- | --- | --- | --- |
| **1** - Ferulic acid | 5.64 ± 0.026 | 5.42 ± 0.012 | 2.79 ± 0.042 | 2.29 ± 0.012 | 2.25 ± 0.021 | 2.42 ± 0.029 |
| **2** - Apocynin | 5.73 ± 0.035 | 5.57 ± 0.031 | 2.87 ± 0.049 | 2.26 ± 0.026 | 2.31 ± 0.023 | 2.46 ± 0.029 |
| **3** - Propyl gallate | 5.72 ± 0.006 | 5.57 ± 0.015 | 2.81 ± 0.044 | 2.31 ± 0.015 | 2.27 ± 0.021 | 2.42 ± 0.006 |
| **4** - Eugenol | 5.70 ± 0.029 | 5.64 ± 0.030 | 3.10 ± 0.030 | 2.54 ± 0.021 | 2.25 ± 0.010 | 2.45 ± 0.057 |
| **5** - Me -dehydrozingerone | 5.76 ± 0.006 | 5.74 ± 0.035 | 5.08 ± 0.052 | 2.31 ± 0.015 | 2.27 ± 0.010 | 2.39 ± 0.012 |
| **6** - Eugenol dimer | 5.74 ± 0.006 | 5.66 ± 0.038 | 3.08 ± 0.010 | 2.46 ± 0.017 | 2.27 ± 0.006 | 2.41 ± 0.040 |
| **7** - Magnolol | 5.74 ± 0.035 | 5.83 ± 0.055 | 5.90 ± 0.023 | 6.20 ± 0.130 | 3.38 ± 0.292 | 2.94 ± 0.010 |
| **8** - Ellagic acid | 5.71 ± 0.012 | 5.53 ± 0.012 | 2.87 ± 0.006 | 2.32 ± 0.025 | 2.33 ± 0.010 | 2.41 ± 0.010 |

**S1 Table. Evolution of pH in Vogel’s medium amended with different phenolic compounds during 0-14 days**

**after inoculation with *F. culmorum* strain FcUK99.**

pH values are expressed as mean (± standard deviation) of three different measurements performed after 0, 1,2,4,8 and 14 days after inoculation.
