# Supplementary material for: Natural Phenolic Inhibitors of Trichothecene Biosynthesis by the Wheat Fungal Pathogen Fusarium culmorum: A Computational Insight into the Structure-Activity Relationship
Source: PLoS One. 2016 Jun 13;11(6):e0157316. doi: 10.1371/journal.pone.0157316 (PMC4905666; doi:10.1371/journal.pone.0157316)
Supplement: S2 Table — (DOCX) [file pone.0157316.s003.docx]

| **Tested ligands** | **%** | **Sites** | **E.F.E.B.^a^** | **E.I.C., Ki^b^** | **Interactions with amino acids** |
| --- | --- | --- | --- | --- | --- |
| Ferulic acid **1** | 2 | c. d.**^c^** | -9.62 | 89.12 nM | Asp100 Glu164 Leu181 Arg182 Asn185 Asp226 Glu233 Arg238 Asp239 Ser242 Leu243 Asn246 PPi700 Mg703 |
|  | 6 | c. d. | -7.78 | 1.99 uM | Ile70 Met73 Tyr93 Thr96 Asp100 Phe157 Arg182 Asn185 Leu187 PPi700 Mg703 |
|  | 13 | 3 | -5.58 | 80.65 uM | Met1 Glu2 Asn3 Phe4 Tyr231 Lys232 Phe234 Asp235 Arg306 |
|  | 8 | 4 | -5.51 | 92.16 uM | Met55 Leu56 Lys57 Arg62 Val98 Ser102 Ser103 Pro126 Trp127 |
|  | 14 | 3 | -5.48 | 96.45 uM | Phe4 Thr6 Tyr231 Phe234 Asp235 Arg306 Lys313 |
|  | 19 | 5 | -5.19 | 157.79 uM | Gln53 Gln54 Leu56 Lys57 Val58 Pro60 Leu63 |
| 3-Hydroxycinnamic acid **9** | 23 | c. d. | -12.65 | 531.99 ρM | Asp100 Glu164 Leu181 Arg182 Asn185 Asp226 Arg238 Asp239 Ile241 Ser242 Leu243 PPi700 Mg702 Mg703 |
|  | 33 | c. d. | -12.08 | 1.41 nM | Asp100 Glu164 Leu181 Arg182 Asn185 Arg238 Asp239 Ile241 Ser242 Leu243 Asn246 PPi700 Mg702 Mg703 |
|  | 8 | 4 | -5.60 | 78.41 uM | Gln53 Gln54 Leu56 Lys57 Val58 Pro60 Leu63 |
|  | 12 | 2 | -5.05 | 200.36 uM | His299 Leu300 Cys301 Asp302 Ala303 Tyr305 Arg306 Leu307 His308 Phe329 Ala333 Ala337 |
| 4-Hydroxycinnamic acid **10** | 75 | c. d. | -13.23 | 199.19 ρM | Asp100 Glu164 Pro178 Leu181 Arg182 Asn185 Asp239 Ile241 Ser242 Leu243 Asn246 PPi700 Mg702 Mg703 |
|  | 1 | c. d. | -7.73 | 2.17 uM | Ile70 Met73 Tyr93 Leu97 Asp100 Phe157 Arg182 Asn185 Leu187 PPi700 Mg703 |
|  | 4 | 3 | -5.59 | 79.22 uM | Met1 Glu2 Asn3 Phe4 Thr6 Phe234 Asp235 Lys313 |
|  | 11 | 3 | -5.56 | 84.50 uM | Phe4 Thr6 Tyr231 Phe234 Asp235 Arg306 Lys313 |
| 2,5-Dimethoxtcinnamic acid **11** | 2 | c. d. | -10.64 | 14.92 nM | Asp100 Glu164 Pro178 Leu181 Arg182 Asn185 Asp226 Glu233 Arg238 Asp239 Ile241 Ser242 Leu243 Asn246 PPi700 Mg702 Mg703 |
|  | 7 | c. d. | -7.28 | 4.64 uM | Met73 Thr96 Leu97 Asp100 Phe157 Arg182 Asn185 Gly186 Leu187 Val191 Met221 PPi700 Mg703 |
|  | 3 | c. d. | -6.61 | 14.17 uM | Tyr93 Thr96 Leu97 Asp100 Leu187 Val191 Met221 Phe291 Tyr295 PPi700 |
|  | 3 | c. d. | -6.28 | 25.1 uM | Met73 Tyr93 Thr96 Leu97 Asp100 Arg182 Gly186 Leu187 Val191 Met221 Val222 Asn225 Tyr295 PPi700 |
|  | 15 | 3 | -5.42 | 106.87 uM | Met1 Glu2 Asn3 Phe4 Thr6 Tyr231 Phe234 Asp235 Lys313 |
|  | 12 | 5 | -5.39 | 111.84 uM | Phe47 Gln52 Gln53 Gln54 Leu56 Lys57 Val58 Pro60 Leu63 |
|  | 14 | 4 | -5.09 | 185.71 uM | Met55 Leu56 Lys57 Arg62 Ser102 Asp104 Pro126 Trp127 |
|  | 16 | 1 | -4.81 | 299.18 uM | Gln68 Gly72 Tyr76 Trp298 Cys301 Aap302 Ala337 Val338 Trp343 |
| 3-Methoxybenzoic acid **12** | 86 | c. d. | -12.19 | 1,16 nM | Asp100 Glu164 Asn185 Glu233 Arg238 Asp239 Ile241 Ser242 PPi700 Mg702 Mg703 |
| 3,4-Dimethoxybenzoic acid **13** | 18 | c. d. | -10.88 | 10.64 nM | Asp100 Glu164 Leu181 Arg182 Asn185 Asp226 Glu233 Arg238 Asp239 Ile241 Ser242 Leu243 PPi700 Mg702 Mg703 |
|  | 30 | c. d. | -5.58 | 81.1 uM | Met73 Tyr93 Arg182 Gly186 Leu187 Val191 Met221 Val222 Asn225 Phe291 Tyr295 PPi700 |

**S2 Table. Docking for protein Q8NIG9**

^a^E.F.E.B. (Estimated Free Energy of Binding)

^b^E.I.C., Ki (Estimated Inhibition Constant, Ki)

^c^c. d. = catalytic domain
