# Supplementary material for: Natural Phenolic Inhibitors of Trichothecene Biosynthesis by the Wheat Fungal Pathogen Fusarium culmorum: A Computational Insight into the Structure-Activity Relationship
Source: PLoS One. 2016 Jun 13;11(6):e0157316. doi: 10.1371/journal.pone.0157316 (PMC4905666; doi:10.1371/journal.pone.0157316)
Supplement: S3 Table — (DOCX) [file pone.0157316.s004.docx]

| **Tested ligands** | **%** | **Sites** | **Hbond** | **Ligands Atom** | **Protein Atom** | **Distance**  **(Å)^a^** | **LogP** | **Dipole Moment**  **(D)** |
| --- | --- | --- | --- | --- | --- | --- | --- | --- |
| Ferulic acid **1** | 2 | c. d^b^ | 5 | O12(OA) | Asn185:2HD2(HD) | 2.118 | 1.70 | 26.467 |
|  |  |  |  | H15(HD) | Asp226:OD1(OA) | 1.887 |  |  |
|  |  |  |  | O11(OA) | Arg238:HE(HD) | **2.299** |  |  |
|  |  |  |  | O11(OA) | Arg238:1HH2(HD) | **2.196** |  |  |
|  |  |  |  | O14(OA) | Leu243:HN(HD) | 1.985 |  |  |
|  | 6 | c. d. | 0 | ………… | ………… | …… |  |  |
|  | 13 | 3 | 4 | H15(HD) | Glu2:O(OA) | 2.023 |  |  |
|  |  |  |  | O14(OA) | Phe4:HN(HD) | 2.093 |  |  |
|  |  |  |  | O10(OA) | Asp235:HN(HD) | 1.925 |  |  |
|  |  |  |  | O11(OA) | Arg306:1HH2(HD) | 1.779 |  |  |
|  | 8 | 4 | 5 | H15(HD) | Met55:O(OA) | 2.269 |  |  |
|  |  |  |  | O12(OA) | Lys57:HN(HD) | 2.147 |  |  |
|  |  |  |  | O10(OA) | Arg62:1HH1(HD) | **1.929** |  |  |
|  |  |  |  | O10(OA) | Arg62:1HH2(HD) | **2.235** |  |  |
|  |  |  |  | O11(OA) | Ser103:HN(HD) | 2.208 |  |  |
|  | 14 | 3 | 7 | O11(OA) | Thr6:HG1(HD) | 2.592 |  |  |
|  |  |  |  | H15(HD) | Tyr231:O(OA) | 1.860 |  |  |
|  |  |  |  | O14(OA) | Asp235:HN(HD) | 2.276 |  |  |
|  |  |  |  | O12(OA) | Arg306:1HH2(HD) | **2.481** |  |  |
|  |  |  |  | O12(OA) | Arg306:2HH2(HD) | **2.391** |  |  |
|  |  |  |  | O10(OA) | Lys313:HZ3(HD) | **1.946** |  |  |
|  |  |  |  | O11(OA) | Lys313:HZ2(HD) | **1.809** |  |  |
|  | 19 | 5 | 3 | O14(OA) | Gln53:2HE2(HD) | 2.271 |  |  |
|  |  |  |  | O10(OA) | Lys57:HZ3(HD) | **2.483** |  |  |
|  |  |  |  | O11(OA) | Lys57:HZ2(HD) | **1.718** |  |  |
| 3-Hydroxycinnamic acid **9** | 23 | c. d. | 2 | H13(HD) | Asp226:OD1(OA) | 1.763 | 1.57 | 19.9876 |
|  |  |  |  | O12(OA) | Leu243:HN(HD) | 2.278 |  |  |
|  | 33 | c. d. | 2 | O12(OA) | Asn246:1HD2(HD) | **2.089** |  |  |
|  |  |  |  | O12(OA) | Asn246:2HD2(HD) | **2.342** |  |  |
|  | 8 | 5 | 3 | O12(OA) | Gln53:2HE2(HD) | **2.233** |  |  |
|  |  |  |  | H13(HD) | Gln53:OE1(OA) | **2.136** |  |  |
|  |  |  |  | O11(OA) | Lys57:HZ2(HD) | 1.880 |  |  |
|  | 12 | 2 | 2 | H13(HD) | His299:O(OA) | 2.116 |  |  |
|  |  |  |  | O12(OA) | Leu307:HN(HD) | 2.021 |  |  |
| 4-Hydroxycinnamic acid **10** | 75 | c. d. | 2 | H13(HD) | Pro178:O(OA) | 2.474 | 1.57 | 18.8772 |
|  |  |  |  | O12(OA) | Asn246:2HD2(HD) | 2.039 |  |  |
|  | 1 | c. d. | 0 | ………… | ………… | …… |  |  |
|  | 4 | 3 | 6 | H13(HD) | Met1:O(OA) | 2.081 |  |  |
|  |  |  |  | O12(OA) | Met1:HN3(HD) | 1.616 |  |  |
|  |  |  |  | H13(HD) | Asp235:OD1(OA) | 2.422 |  |  |
|  |  |  |  | O10(OA) | Lys313:HZ2(HD) | **1.902** |  |  |
|  |  |  |  | O11(OA) | Lys313:HZ2(HD) | **2.585** |  |  |
|  |  |  |  | O11(OA) | Lys313:HZ3(HD) | **1.848** |  |  |
|  | 11 | 3 | 5 | O10(OA) | Thr6:HG1(HD) | 2.515 |  |  |
|  |  |  |  | H13(HD) | Tyr231:O(OA) | 1.895 |  |  |
|  |  |  |  | O12(OA) | Asp235:HN(HD) | 2.221 |  |  |
|  |  |  |  | O10(OA) | Lys313:HZ2(HD) | **1.852** |  |  |
|  |  |  |  | O11(OA) | Lys313:HZ3(HD) | **1.962** |  |  |

**S3 Table. H-bond interaction of tested ligands-protein, logP and Dipole Moment of the ligands.**

| 2,5-Dimethoxycinnamic acid **11** | 2 | c. d. | 0 | ………… | …………… | …… | 2.22 | 16.6715 |
| --- | --- | --- | --- | --- | --- | --- | --- | --- |
|  | 7 | c. d. | 0 | ………… | …………… | …… |  |  |
|  | 3 | c. d. | 0 | ………… | …………… | …… |  |  |
|  | 3 | c. d. | 2 | O14(OA) | Thr96:HG1(HD) | 2.268 |  |  |
|  |  |  |  | O11(OA) | Arg182:2HH2(HD) | 1.852 |  |  |
|  | 15 | 3 | 3 | O12(OA) | Phe4:HN(HD) | 2.047 |  |  |
|  |  |  |  | O10(OA) | Lys313:HZ2(HD) | **1.869** |  |  |
|  |  |  |  | O11(OA) | Lys313:HZ3(HD) | **1.887** |  |  |
|  | 12 | 5 | 2 | O11(OA) | Lys57:HZ2(HD) | **1.846** |  |  |
|  |  |  |  | O10(OA) | Lys57:HZ3(HD) | **2.276** |  |  |
|  | 14 | 4 | 3 | O14(OA) | Lys57:HN(HD) | **2.220** |  |  |
|  |  |  |  | O10(OA) | Arg62:1HH1(HD) | 2.117 |  |  |
|  |  |  |  | O12(OA) | Ser102:HG(HD) | 2.329 |  |  |
|  | 16 | 1 | 2 | O14(OA) | Gln68:1HE2(HD) | 2.133 |  |  |
|  |  |  |  | O11(OA) | Val338:HN(HD) | 2.212 |  |  |
| 3-Methoxybenzoic acid **12** | 86 | c. d. | 0 | ………… | ………… | …… | 1.35 | 12.6355 |
| 3,4-Dimethoxybenzoic acid **13** | 18 | c. d. | 2 | O10(OA) | Asn185:2HD2(HD) | 1.854 | 1.49 | 37.0086 |
|  |  |  |  | O12(OA) | Leu243:HN(HD) | 1.685 |  |  |
|  | 30 | c. d. | 2 | O9(OA) | Arg182:2HH2(HD) | 1.860 |  |  |
|  |  |  |  | O8(OA) | Tyr295:HH(HD) | 2.377 |  |  |

^a^in bold cross-bridge H-bond interactions with the same aa

^b^c. d.= catalytic domain
